# Sex differences in AMPA receptor trafficking proteins

**DOI:** 10.1186/s13293-026-00849-1

**Published:** 2026-02-13

**Authors:** Mia Y. Roberts, Lisa A. Briand

**Affiliations:** 1 Department of Psychology and Neuroscience, Temple University, Weiss Hall, 1701 North 13th Street, Philadelphia, 19122 PA USA; 2 Neuroscience Program, Temple University, Philadelphia, PA 19122 USA

**Keywords:** Sex differences, AMPA receptor trafficking, Trafficking proteins

## Abstract

AMPA receptors are a type of ionotropic glutamate receptor that is important for fast excitatory neurotransmission. healthy brain function. Glutamate signaling is regulated, in part, by the trafficking of glutamate receptors in and out of the synapse. Multiple different trafficking and auxiliary proteins govern this process. Disruptions in this trafficking are linked to various psychiatric diseases, including schizophrenia, major depressive disorder. Glutamate, the primary excitatory neurotransmitter, is crucial for synaptic plasticity and and substance use disorder. Moreover, the incidence and symptomology of these psychiatric diseases impact males and females differently. Despite these epidemiological sex differences, very little research has considered the influence of biological sex on glutamatergic trafficking. Here, we review the current literature on glutamate trafficking proteins for AMPA receptors, most of which have mainly utilized male rodents and cell cultures. The following proteins were explored for AMPA receptors: GRIP, PICK1, NSF, SAP97, AKAP79/150, Protein 4.1 N, and PSD-95. Overall, these studies revealed that our fundamental understanding of glutamate trafficking is based almost completely on studies performed in male animals, and the assumption that the same mechanisms govern AMPAR trafficking in females may not be correct. To fully grasp how these proteins are impacted in disease models, it’s crucial to first understand the baseline sex differences. This is especially important if we want to investigate new research avenues for treating diseases that affect each sex differently.

## Introduction

Converging lines of evidence suggest that the trafficking of glutamate receptors, which regulates receptor surface expression and synaptic strength, may play a critical role in mediating normal and pathological brain function [1–3]. Disruptions in glutamatergic neurotransmission are central to multiple psychiatric diseases, including schizophrenia, Alzheimer’s disease (AD), major depressive disorder (MDD), and substance use disorders (SUDs) [4–7]. Moreover, these diseases impact males and females differently, with the incidence of schizophrenia and SUDs being higher in males, while AD and MDD are more prevalent in females [8–11]. However, very little is known about the influence of biological sex on glutamate trafficking both at baseline and in response to stimuli. This is due, in part, to the historical focus in preclinical research on male subjects [12, 13].

Synaptic plasticity, or the ability of a neuron to alter the strength of its connections, is implicated in learning, memory, and the development of neural circuits essential for optimal brain function [14]. Various forms of activity-dependent plasticity exist, both short- and long-term, facilitating adaptations within the brain [15]. The two central forms of long-term activity-dependent synaptic plasticity are long-term potentiation (LTP) and long-term depression (LTD). LTP is the strengthening of synaptic connections, while LTD is the weakening of connections [16]. Synaptic plasticity within the glutamate system is governed, in part, by the trafficking of receptors to and from the synaptic membrane. A multitude of different proteins are involved in this trafficking, which impact glutamatergic synaptic function, learning, and drug-seeking behaviors in rodents (Pushkin et al., 2023; Wang et al., 2014; Lee & Messing, 2011). More specifically, plasticity within the glutamate system involves the cycling of AMPA (α-amino-3-hydroxy-5-methyl-4-isoxazole propionic acid) receptors (AMPARs) and NMDA (N-methyl-D-aspartate) receptors (NMDARs) in and out of synaptic and extrasynaptic sites. Receptors are trafficked in and out of the surface through processes known as exocytosis and endocytosis. Additionally, auxiliary and trafficking proteins assist in the lateral movement of these receptors throughout the processes [17–19]. This dynamic process allows the cell to adjust the number of receptors in response to changing environmental conditions. It is important to note that most, if not all, of the research examining the role of these trafficking proteins in glutamate receptor trafficking has been conducted using male animals or cell cultures, and it is unknown whether these proteins play a similar role in females. This review will focus on the impact of biological sex on AMPA receptor protein trafficking and its ability to modify receptor trafficking and synaptic plasticity.

## AMPA receptor trafficking

AMPA receptors are ionotropic glutamatergic receptors that are ligand-gated and thought to mediate fast excitatory neurotransmission [20, 21]. These receptors can be further classified into their subunits. The AMPAR GluA subunits (GluA1-4) randomly combine to form tetramers. The composition of AMPARs can confer different properties. Specifically, the calcium permeability of AMPARs is dependent on the presence or absence of the GluA2 subunit. AMPARs containing a GluA2 subunit are impermeable to calcium, while the absence of the subunit results in calcium permeability and is altered in response to neuronal stress and neurological diseases [22]. It is recognized that the trafficking of AMPARs is essential for both LTP and LTD. The subunit composition of AMPARs is pivotal in facilitating the scaling up and scaling down of synaptic transmission [23]. The method through which AMPARs change subunit composition involves utilizing various trafficking and auxiliary proteins that assist in receptor trafficking and function.

Accumulating research suggests a role for AMPAR trafficking and subunit composition in activity-dependent LTP [24–27]. LTP can occur by increasing the number of total AMPARs at the synapse, typically via GluA2 AMPAR insertion, as the majority of AMPARs contain GluA2 [28–31]. A widely proposed model of AMPARs’ role in LTP in males involves the insertion of GluA2-lacking AMPARs during LTP induction, which have a greater conductance and therefore can strengthen the synapse without the addition of more receptors [32–35]. However, evidence suggests that this may not always be the case, as pharmacological antagonism of GluA2-lacking AMPARs does not always impair LTP, suggesting that GluA2-lacking AMPAR insertion may not be necessary for LTP induction [36]. While this finding may seem inconsistent with the proposed model, one explanation may be due to differences in the roles of glutamate receptor trafficking proteins. Glutamate receptor trafficking and the proteins involved in this regulation are important for neurotransmission and, possibly, downstream physiological effects like learning, memory, and reward signaling [35, 37]. Regulation of the number of AMPARs at the postsynaptic density is a fundamental mechanism underlying synaptic plasticity. The C-termini of AMPAR subunits bind to different trafficking and auxiliary proteins, leading to distinct trafficking patterns [38].

These are well-defined mechanisms, but research has predominantly been conducted in male animals. Preclinical work has reported sex differences within the glutamatergic system on both the pre- and post-synaptic sides across various brain regions, which could contribute to sex differences present in the mentioned psychiatric diseases like SUDs [39–44]. For instance, in the nucleus accumbens, female mice exhibit a larger readily releasable pool and higher spine density [39, 45]. On the postsynaptic side, a similar trend is present, as females measure a higher AMPA/NMDA ratio and larger mEPSC amplitude than male mice [39]. Overall, females exhibit higher presynaptic and postsynaptic glutamatergic transmission in the nucleus accumbens. These findings may be influenced by sex-specific differences in the expression or the functional roles of the proteins involved in glutamate receptor trafficking in females. However, little is known about how biological sex influences the functional role of trafficking proteins in these processes. Accordingly, the following sections will discuss the roles of these proteins in AMPAR trafficking and synaptic plasticity, based on what is known in males and, where possible, in females.

### The influence of sex on glutamate signaling

Various studies have reported sex differences in glutamatergic transmission throughout the brain. In humans, proton magnetic resonance spectroscopy (MRS) studies have reported higher glutamate levels in the parietal gray matter in males compared to females [46]. However, when examining specific brain regions, the opposite is seen in the striatum and cerebellum [47]. It is important to note that measuring total glutamate levels broadly does not specifically reflect neurotransmission, as glutamate is also a metabolic byproduct in most brain cells [48]. Therefore, observed sex differences in these studies might stem from differences in metabolic processes rather than neurotransmission. Nevertheless, more precise measurements of glutamate levels in rodents, in which sex differences are present across various brain regions, can be attributed to neurotransmission. Females have been found to have higher levels of glutamate receptors and increased levels of glutamate in areas such as the hippocampus and the medial preoptic area [49–51]. Additionally, levels of glutamate in females fluctuate throughout their estrous cycle and are region specific [41, 49, 50]. Therefore, it is of utmost importance to consider sex differences in glutamate receptor trafficking, as this is essential for understanding behavioral and molecular differences between males and females. Identifying the receptors affected by glutamate transmission differences between sexes is vital for understanding the nature of the signal. While a behavior might appear similar in both sexes, the mechanisms driving that behavior can vary. This understanding allows researchers, for example, to create more effective pharmacotherapies tailored to each sex.

While it may seem that there are clearly defined mechanisms driving the trafficking of AMPAR subunits and receptors, it is important to note that the majority of work that has established these mechanisms was done in exclusively male animals or in cell culture, where biological sex was not considered. Whether these proteins function similarly in female rodents remains yet to be determined, as sex is a biological factor that is heavily understudied. The use of males as the standard and the expectation that females will respond similarly to males can lead to the misinterpretation of data. Historically, females have been excluded from most basic biological studies, in part due to the fluctuating estrous cycle and the potential for ovarian hormones to induce structural changes in neural plasticity [52, 53]. Yet, recent papers have debunked this idea to show that this is not true and that female rodents are not more variable than male rodents [54–56]. Understanding the impact of trafficking proteins in females is essential for enhancing our comprehension of their underlying biological processes.

## GluA1 trafficking proteins

**Fig. 1:**
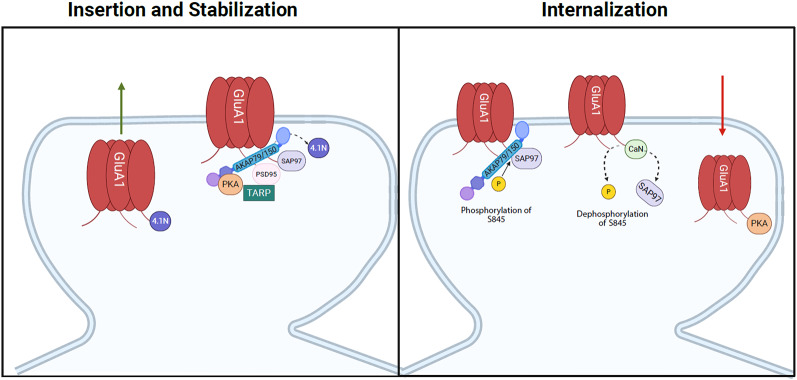
Postsynaptic regulation of GluA1-containing AMPARs by SAP97, PSD-95, TARPs, 4.1 N, and AKAP79/150*.* 4.1 N mediates the insertion of GluA1-containing AMPARs, whereas SAP97 stabilizes the receptors to the plasma membrane. Transmembrane AMPAR Regulatory Proteins (TARPs) assist PSD-95 in anchoring and clustering receptors at the cell surface. Additionally, AKAP79/150 facilitates phosphorylation of serine 845 on the C-terminal tail of the GluA1 subunit and binds PKA. In contrast, when AKAP79/150 interacts with calcineurin (CaN), it dephosphorylates S845, thereby promoting receptor internalization

Multiple trafficking and auxiliary proteins are involved in the regulation of GluA1-containing AMPARs. These are important for either the insertion or internalization of GluA1. Despite their distinct roles, these proteins work in tandem to regulate trafficking and synaptic plasticity. The trafficking proteins that exclusively regulate the insertion and anchoring of the GluA1 subunit are synapse-associated protein 97 (SAP97), postsynaptic density 95 protein (PSD-95), and protein 4.1 N, while A kinase anchoring protein 79 (AKAP150 in rodents) (AKAP79/150) regulates the internalization of GluA1 (Fig. 1). The following section will discuss the functional role of the mentioned proteins in AMPAR trafficking and synaptic plasticity in males and, when possible, in females.

### GluA1: synapse-associated protein 97 (SAP97)

The AMPAR GluA1 subunit has been implicated in synaptic plasticity and neuronal development [57, 58]. A prime example of this is SAP97, a scaffolding protein that binds exclusively to the PDZ domain of GluA1 subunits and is involved in anchoring and inserting GluA1-containing AMPARs at the synapse (Fig. 1) [59–61]. Overexpression of SAP97 increases the amount of GluA1-containing AMPARs surface expression [62]. In contrast, the downregulation of SAP97 decreases GluA1 expression at the synapse [63]. Specifically, SAP97 is involved in the early trafficking of GluA1 to the membrane and maintenance of extrasynaptic AMPAR at the surface. Only a small proportion of GluA1 receptors engage with SAP97 at the synapse, as it is believed that SAP97 primarily interacts with GluA1 in the early stages of the secretory pathway, specifically at the endoplasmic reticulum or Golgi apparatus [64]. These findings suggest that SAP97 is necessary for anchoring GluA1-containing AMPARs to the plasma membrane but is not essential for their internalization.

SAP97 is a key regulator of activity-dependent synaptic plasticity, demonstrating its pivotal role in neurotransmission processes. SAP97 exists in two isoforms, αSAP97 and βSAP97. The function of αSAP97 is believed to involve the regulation of AMPAR-mediated transmission through an activity-independent pathway, while βSAP97 operates in an activity-dependent manner that relies on the utilization of CAMKII [58]. Disruption of these isoforms leads to impaired LTP and enhanced LTD [65]. Furthermore, overexpression of αSAP97 increases the synaptic pool of AMPAR, leading to LTP occlusion. In contrast, overexpression of βSAP97 directs AMPARs at the perisynapse and prevents LTP induction [66]. These findings suggest that both isoforms are necessary for the insertion of GluA1-containing AMPARs and synaptic plasticity but mediate them through different mechanisms. Moreover, this highlights the role of SAP97 in directly modulating the function of synaptic GluA1-containing AMPARs. Overexpression of SAP97 leads to an increase in mEPSC frequency and dendritic spine enlargements, indicating that SAP97 impacts both presynaptic and postsynaptic transmission [62]. More electrophysiological data suggests that SAP97 controls synaptic strength by regulating the concentration of GluA1-containing AMPAR, thereby impacting their ability to release glutamate presynaptically [65]. The interaction between SAP97 and GluA1 showcases this protein’s involvement in the insertion of GluA1-containing AMPARs and in synaptic activity.

Work examining sex as a biological variable in the trafficking of GluA1 subunits is limited. In clinical populations, there is evidence that SAP97 has sexually dimorphic effects, as a single nucleotide polymorphism (SNP) in the SAP97 gene is associated with an increased risk for schizophrenia in males but not females [67, 68]. In rodent models, conditional knockout of SAP97 in the hippocampus of mice leads to sex specific behavioral phenotypes, with males exhibiting deficits in novel object recognition and females exhibiting locomotor and motor learning deficits in the open field and rotarod tasks [69, 70]. This could suggest that the role of SAP97 in AMPAR trafficking in the hippocampus is different in males and females or that AMPAR trafficking plays a sex-specific role in these behaviors. However, physiological experiments examining SAP97’s functional role in AMPA trafficking and synaptic plasticity in females are necessary to determine which of the two scenarios is occurring.

### Protein 4.1 N

The cytoskeletal protein 4.1 N is thought to facilitate both the insertion and stabilization of GluA1-containing AMPARs influencing their surface expression, while SAP97 primarily contributes to the stabilization of GluA1-containing AMPARs (Fig. 1) [71]. When 4.1 N binds to GluA1, it increases the insertion of GluA1-containing receptors [63]. In contrast, when 4.1 N is knocked down, the frequency of GluA1 insertion is reduced [71, 72]. Additionally, the downregulation of 4.1 N decreases GluA1 trafficking, which indicates that 4.1 N is vital for GluA1 insertion at the surface [63]. The mechanistic differences highlight the distinct roles of 4.1 N and SAP97 for the insertion of GluA1-containing AMPAR. Despite the distinct roles, both 4.1 N and SAP97 are critical for GluA1-containing AMPAR insertion, as removal of either protein disrupts the process [63]. This suggests that these redundant processes are important for regulating GluA1-containing AMPAR insertion.

Due to its important role in regulating the trafficking and recycling of GluA1 receptors, it is reasonable to posit that 4.1 N also impacts synaptic plasticity. When LTP is chemically induced, the function 4.1 N shifts from solely stabilizing GluA1-containing AMPAR receptors to also supporting SAP97-mediated insertion, thereby boosting LTP expression [63]. This indicates that trafficking proteins do not operate independently but work together to maintain normal function. When 4.1 N is knocked down, LTP is significantly reduced after induction, suggesting that 4.1 N is necessary for LTP expression [72]. Additionally, after induction, hippocampal LTP is blunted when GluA1–4.1 N binding is disrupted [73]. Moreover, during LTP, GluA1 serines 816 and 818 are phosphorylated, resulting in increased binding of 4.1 N to GluA1 and insertion of GluA1 [63, 74]. These studies suggest that 4.1 N is an essential protein for both intercellular transport, insertion, and LTP expression. However, further research is needed to better elucidate the role of protein 4.1 N in synaptic plasticity.

In contrast to the potential sex specific role for SAP97, manipulation of hippocampal 4.1 N leads to similar effects in both sexes. Reducing 4.1 N expression in the dentate gyrus granule neurons leads to a reduction in AMPAR eEPSC and NMDAR eEPSC amplitude, and reduces dendritic spine density in both males and females, indicating that biological sex may not play a role in the effects of 4.1 N on glutamatergic synapse function [75]. However, these findings are limited to a single publication in a single brain region, and further work is needed to determine whether 4.1 N plays a similar role across biological sex throughout the brain. Since 4.1 N and SAP97 both facilitate GluA1 insertion, future research should examine how hippocampal 4.1 N influences behavior, such as performance on learning tasks, across both sexes. More physiological work in females is also necessary to further draw conclusions about 4.1 N’s role in synaptic plasticity. Studies like these would provide insights into the protein’s functional role in synaptic plasticity, AMPAR trafficking, and behavior.

### Postsynaptic density protein-95 (PSD-95)

PSD-95 is a scaffolding protein in the membrane-associated guanylate kinase (MAGUK) family, essential for regulating the maturation of synapses through its interactions with NMDA and AMPAR [76–78]. Mechanistically, PSD-95 is heavily implicated in anchoring and clustering GluA1-containing AMPARs to the synaptic surface, where overexpression of PSD-95 in hippocampal neurons enhances the localization of GluA1-containing AMPARs at the synapse (Fig. 1) [78–80]. PSD-95 facilitates these effects through protein palmitoylation, where the addition of the fatty acid palmitate influences the retention of AMPARs [80, 81]. The palmitoylation of PSD-95 increases binding between the scaffolding protein and GluA1 subunit, where reduction of palmitoylation of PSD-95 leads to decreased synaptic clustering of GluA1 and PSD-95 [80, 82]. The regulation of GluA1-AMPAR via PSD-95 also occurs through its interaction with stargazin, a protein in the TARP (transmembrane AMPAR regulatory proteins) family that interacts with all AMPAR subunits, including GluA1, GluA2, GluA3, and GluA4 [59]. Overexpression of stargazin significantly increases extrasynaptic GluA1-containing AMPARs, but this increase is prevented when PSD-95 levels are kept constant, which suggests a direct interaction between the two proteins in synaptic AMPAR regulation [83]. The research on PSD-95 demonstrates an important role in regulating AMAPR subunit composition, particularly its involvement in the insertion and synaptic expression of GluA1-containing receptors.

The function of PSD-95 is unique compared to other scaffolding proteins, as it is expressed after synaptogenesis and is responsible for the maturity and stabilization of excitatory synapses [58, 78, 84, 85]. Thus, acute knockdown and a point mutation that inactivates PSD-95 impair spine growth, which in turn can impact synaptic plasticity [86–88]. Overexpression studies have demonstrated that excess PSD-95 increases AMPAR EPSCs and occludes LTP [89]. The opposite is also true, as functional mutations of PSD-95 enhance LTP, irrespective of stimulation frequency [90]. Further, PSD-95 overexpression can enhance LTD, possibly due to elevated basal activity of AMPARs in hippocampal cells [79, 91, 92]. In contrast, knockdown of PSD-95 impairs LTD, indicating that PSD-95 presence is important for inducing LTD [86]. Overall, these findings imply a role for PSD-95 in AMPA-mediated synaptic activity.

Extensive research has investigated how sex influences PSD-95 expression and GluA1 regulation. In ovariectomized female mice, levels of hippocampal PSD-95 and GluA1 dropped two weeks post-surgery, but by four weeks, GluA1 levels increased again [93]. In contrast, orchiectomized male mice demonstrated lower hippocampal levels of both PSD-95 and GluA1 [93]. This suggests that gonadal hormones directly influence PSD-95 and GluA1 regulation in both sexes, although the underlying mechanisms vary. Furthermore, in female mice, inhibiting aromatase, an enzyme that converts androgens into estrogens, resulted in a notable reduction of PSD-95 levels in the hippocampus [94]. This result highlights estrogen’s role in regulating PSD-95 and possibly GluA1 in females. Interestingly, removal of PSD-95 in mice decreases preference for ethanol during conditioned place preference for both sexes, suggesting that while PSD-95 may mediate addiction-like behaviors, at least for conditioned place preference, this is not done in a sex-dependent manner [95]. These findings indicate that gonadal hormones may differentially affect PSD-95 expression and GluA1 trafficking in the hippocampus of male and female mice.

### A kinase anchoring protein 79/150 (AKAP79/150)

AKAP79/150 is a regulatory protein that anchors kinases and phosphatases. In AMPAR trafficking, AKAP79/150 assists in the trafficking of GluA1-containing AMPARs through its interaction with phosphatases like calcineurin and kinases, such as protein kinase A (PKA), to mediate phosphorylation of serine 845 (S845) residue on GluA1 (Fig. 1) [96, 97]. AKAP79/150’s interaction with PKA promotes phosphorylation of S845, thereby leading to receptor insertion [96, 98]. AKAP79/150 facilitates phosphorylation through its interaction with SAP97, which causes protein kinase A (PKA) to bind to the GluA1 subunit [98, 99]. This interaction results in the phosphorylation of S845 on GluA1, and thus, insertion of GluA1-containing AMPARs. In contrast, AKAP79/150’s interaction with calcineurin dephosphorylates S845, leading to internalization of GluA1-containing AMPARs [97, 100]. When calcineurin anchoring to AKAP79/150 is disrupted, phosphorylation of S845 increases and removal of GluA1 from the synapse is impaired [100, 101]. Collectively, these findings showcase AKAP79/150’s bidirectional role in GluA1-containing AMPAR trafficking.

S845 phosphorylation by PKA is essential for GluA1-containing AMPAR insertion and is therefore a key regulator of glutamatergic synaptic plasticity. During LTD, GluA1-containing AMPARs are transiently recruited to the synapse following S845 phosphorylation, which is mediated through AKAP79/150 anchoring of PKA [100, 102]. This emphasizes the role of AKAP79/150 in activity-dependent synaptic plasticity. The expression of AKAP79/150 is sufficient enough to modulate GluA1-containing AMPAR currents in hippocampal cells [103]. Disruption of AKAP-PKA anchoring reduces GluA1-containing AMPAR insertion on the membrane and increases GluA2-containing AMPARs [104]. Furthermore, this disruption not only inhibits the induction of LTP but also primes the synapse for LTD signaling, thereby promoting the removal of the GluA2-containing AMPARs [104]. Likewise, disrupting AKAP-calcineurin interaction impairs LTD, but enhances LTP due to enhanced GluA1 S845 phosphorylation [101]. These findings emphasize the importance of AKAP-PKA and AKAP-calcineurin interactions in the synaptic scaling of GluA2-lacking/GluA2-containing AMPARs during normal plasticity. To date, there is no work directly examining sex as a biological factor in the role of AKAP79/150. Therefore, it is unknown whether the function is different in females, highlighting the work that needs to be done.

## GluA2 trafficking proteins

**Fig. 2:**
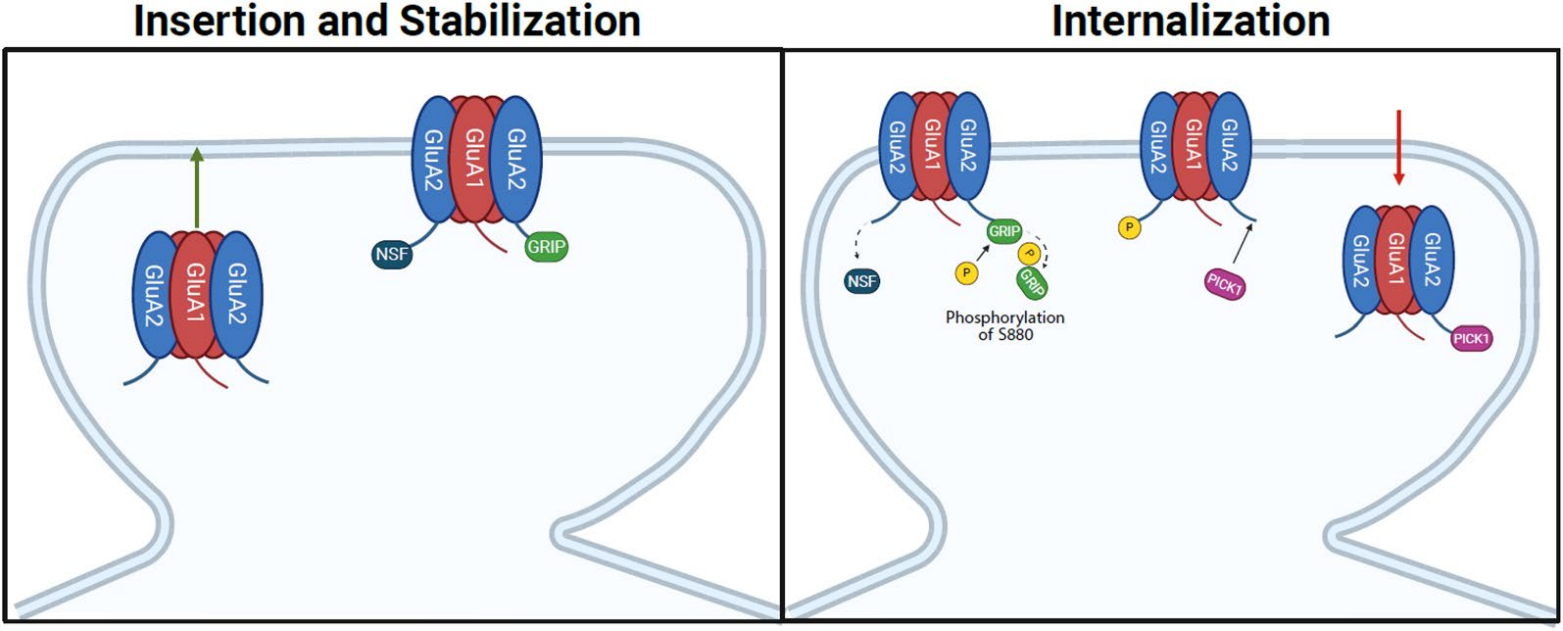
Postsynaptic regulation of GluA2-containing AMPARs by GRIP1, NSF, and PICK1. GRIP1 and NSF facilitate the insertion of GluA2-containing AMPARs unto the plasma membrane. Activity-dependent internalization is mediated by the phosphorylation of serine 880 on the C-terminal tail of the GluA2 subunit, which leads to GRIP dissociation from the membrane, and PICK1 binds to the GluA2 subunit, initiating the internalization of GluA2-containing AMPARs

Similar to the GluA1 subunit, the movement of GluA2-containing AMPARs is regulated by trafficking proteins with specificity for the subunit. The trafficking proteins that exclusively regulate the insertion and stabilization of the GluA2 subunit in AMPAR trafficking include glutamate receptor-interacting protein 1/2 (GRIP1/2) and N-Ethylmaleimide–sensitive fusion protein (NSF), while protein interacting with C kinase 1 (PICK1) is involved in internalizing the subunit (Fig. 2). This section will discuss the functional role of the mentioned proteins in AMPAR trafficking and synaptic plasticity in males, and when possible, in females.

### Glutamate receptor interacting protein (GRIP1) and AMPA receptor-binding protein (ABP/GRIP2)

GRIP1 and AMPA receptor-binding protein (ABP/GRIP2) are two closely related PDZ domain-containing proteins involved in the insertion of GluA2-containing AMPARs (Fig. 2) [105, 106]. The PDZ domain of GRIP/ABP binds to the intracellular C terminus of the GluA2 subunit of AMPARs and anchors the receptor to the cell membrane, which is essential for activity-dependent synaptic plasticity throughout the brain [105]. Additionally, phosphorylation of the serine 880 residue (S880) on GluA2 prevents GRIP/ABP from binding to the subunit (Fig. 2) [107]. Moreover, in hippocampal slices, altering GluA2 S880 phosphorylation through specific point mutations disrupts LTD. Mimicking GluA2 S880 phosphorylation leads to the exclusion of mutated receptors from the synapse and partially occludes LTD, while preventing GluA2 S880 phosphorylation reduces LTD [108]. These findings highlight GluA2’s regulatory mechanisms. If GluA2 is mutated to lack a PDZ binding site or the PDZ interaction with C-terminal peptides is competitively inhibited, there is a significant reduction or complete elimination of GluA2-containing AMPARs that accumulate at the synapse [105, 109, 110]. Based on these overall findings, it is apparent that GRIP/ABP is involved in AMPAR trafficking and localization.

GRIP1/ABP is essential for activity-dependent synaptic plasticity in the hippocampus, cerebellum, nucleus accumbens, and retina [111–114]. Electrophysiological data have shown that deletion of GRIP1 results in blunted LTD [111, 115]. This may be due to the deletion slowing AMPAR recycling [116]. Furthermore, we see that GRIP1 knockout mice exhibit impaired LTP, highlighting GRIP’s role in activity-dependent synaptic plasticity [117]. This indicates that the interaction between GluA2-GRIP/ABP is essential for the incorporation of GluA2-containing AMPARs at the synapse and plays a role in initiating and preserving LTD and LTP in the synapse.

Behavioral sex differences are not observed following GRIP disruption. Hippocampal GRIP1 KD induces deficits during novel object recognition in both sexes, suggesting that this response may not vary between males and females [117]. However, as the data were collapsed across sex and the sample size for each sex is not reported, any potential sex differences may be masked. Prefrontal GRIP KD in mice potentiates cue-induced cocaine seeking and motivation for cocaine in both sexes, but not during food self-administration, suggesting that this effect is specific to cocaine [118]. In this study, sex is also collapsed, but the sample sizes for male and female mice are adequately powered and unpublished analyses from our lab demonstrate that GRIP knockdown increased cocaine seeking and motivation in both sexes. Together, these studies suggest that the functional role of GRIP may be the same across sexes. More studies that treat sex as a biological variable are necessary to elucidate the behavioral impact of GRIP KD. Additional research is needed to determine whether the physiological function of GRIP in females is comparable to that in males, particularly given the evidence that GRIP does not have sex-specific behavioral effects on learning and reward-related behaviors. Alternatively, although the same behaviors were observed after GRIP KO, the underlying mechanisms may vary between sexes.

### Protein interacting with C kinase 1 (PICK1)

PICK1 is another key scaffolding protein involved in the trafficking of GluA2-containing AMPARs. It has a PDZ domain that binds to numerous membrane proteins with a PDZ binding motif, such as GluA2, and a BAR domain implicated in lipid binding [119, 120]. Specifically, PICK1 binds to the GluA2 subunit and internalizes GluA2-containing AMPARs, especially within the first few minutes following LTP induction (Fig. 2) [35, 37, 121, 122]. Overexpression of PICK1 is associated with increased synaptic insertion of GluA2-lacking AMPARS [31, 37]. This suggests that PICK1 is vital in regulating AMPAR subunit composition and synaptic transmission. PICK1 binding to GluA2-containing AMPARs is induced by the phosphorylation of GluA2 S880, which leads to the dissociation of GRIP/ABP, thus allowing PICK1 to bind and internalize the receptor [31, 33, 105]. This can lead to long-term changes within the cell [37]. PICK1 facilitates AMPAR internalization and inhibition of receptor recycling [31, 123]. Further, PICK1 facilitates the trafficking rate through the utilization of endocytic compartments [124–126]. Additionally, PICK1 binds to activated PKCα, targeting synapses and resulting in the phosphorylation of S880, thereby facilitating the endocytosis of GluA2-containing AMPAR and modulating synaptic transmission [108, 127].

PICK1 is also known to play a role in activity-dependent synaptic plasticity, where the interaction between PICK1 and GluA2 regulates the induction of LTP and LTD. Specifically, when PICK1 is knocked down, LTP is inhibited; when PICK1 is overexpressed, it is occluded [121]. PICK1 is also responsible for cerebellar LTD, where blocking PDZ binding between PICK1 and GluA2 attenuates LTD in Purkinje cells, suggesting that binding of PICK1 to GluA2 is required for the LTD induction in cerebellar cells [128, 129]. Taken together, these observations suggest that PICK1 plays a significant role in receptor trafficking and synaptic plasticity.

In contrast to GRIP, prefrontal KD of PICK1 produces sex-specific responses on cocaine seeking. Specifically, prefrontal PICK1 KD attenuates cue-induced cocaine seeking in male mice and potentiates it in females [130]. This effect in females is reversed when tested in ovariectomized female mice, suggesting that not only does prefrontal PICK1 seem to play different roles in males and females, but sex hormones may mediate these effects [131]. PICK1 also seems to play a sex-specific role in schizophrenia, as a recent study found that this effect may be specific to males [132]. Specifically, a point mutation on the BAR domain that is associated with schizophrenia in humans causes deficits in hippocampal-dependent reversal learning in male but not female mice [133]. Additionally, decreased expression of hippocampal and prefrontal GluA1 and GluA2 was observed in male mice only, suggesting a role for the BAR domain in prefrontal AMPAR clustering in males only [120, 133]. Overall, the mentioned studies imply that PICK1 plays a sex- and region-specific role in the brain. As suggested by the PICK1 ovariectomy study, perhaps these roles may be mediated by gonadal hormones or higher gonadal organizational effects. Another possible explanation for these results is that PICK1 likely participates in a sex-specific pathway, and its removal produces sex-dependent differences in the behavior. These studies indicate that sex is an important variable to consider when investigating these proteins’ function, and further research is required to gain a better understanding of the fundamental aspects of glutamatergic trafficking in females.

### N-ethylmaleimide sensitive fusion protein (NSF)

NSF is an ATPase involved in membrane fusion and also assists in regulating GluA2-containing AMPAR surface expression [26, 134–136]. While GRIP anchors AMPARs to the cell surface and PICK1 assists in internalization, NSF is involved in the maintenance of synaptic GluA2-containing AMPARs during insertion (Fig. 2). When NSF-GluA2 binding is inhibited, it decreases AMPAR surface expression and reduces AMPAR-mediated synaptic transmission, highlighting its importance in AMPAR trafficking [26, 137]. The process through which NSF inhibits the internalization of GluA2-containing AMPARs involves preventing GluA2 from interacting with AP2, an endocytic adaptor protein that facilitates the internalization of the receptor [138]. Moreover, NSF inhibits the interaction of PICK1 with GluA2, thereby hindering PICK1-mediated internalization and potentially obstructing GluA2-lacking AMPAR insertion [139].

It is well known that NSF is involved in regulating synaptic plasticity due to its interaction with GluA2 [26, 134, 137–143]. When the interaction between NSF and GluA2 is blocked, it leads to blunted LTD in the hippocampus and decreased AMPAR-mediated excitatory postsynaptic currents [26, 140]. Additionally, the NSF inhibitor, N-ethylmaleimide, blocks LTP induction [144]. These findings suggest that NSF is also involved in activity-dependent synaptic transmission, which may partly be due to the role NSF plays in AMPAR trafficking alongside the other GluA2-trafficking proteins. To date, no research has directly investigated sex as a biological factor in the role of NSF. Therefore, it remains unclear whether the function differs in females, highlighting the work that needs to be done.

## Auxiliary trafficking proteins

The auxiliary trafficking proteins discussed up until now are not the only ones involved in AMPAR trafficking. In fact, there are other key AMPAR interacting proteins that work alongside the previously mentioned proteins to regulate and maintain AMPAR trafficking and function. This section will discuss the cornichon (CNIH) and transmembrane AMPAR regulatory protein (TARP) families and their major roles in trafficking and synaptic plasticity. Additionally, if available, we will discuss how sex, as a biological factor, impacts these proteins and their function.

### Cornichons (CNIH2/3)

Additional auxiliary proteins involved in regulating AMPAR trafficking include cornichons 2 and 3 (CNIH2/3), which are transmembrane chaperone proteins involved in the insertion of GluA1-containing AMPARs (Fig. 1) [145–148]. As CNIH2 is expressed at higher levels in the hippocampus, it has been the focus of the majority of work on CNIH, either alone or in combination with CNIH3 [149]. Removal of CNIH2/3 increases internalization of GluA1-containing AMPARs and impairs AMPAR-evoked EPSCs, an effect not seen following knocking out CNIH3, suggesting that both CNIH2/3 are critical for normal AMPAR function [146]. While there are no reported sex differences related to CNIH2-induced alterations in AMPAR trafficking and signaling, sex differences in the role of CNIH3 on synaptic plasticity and spatial memory have been found. Removal of CNIH3 increases expression of GluA2-containing AMPARs and attenuates LTP in females [145]. Further, removing CNIH3 impairs short-term spatial memory in females in the Barnes maze, an effect that’s reversed when CNIH3 is overexpressed [145]. These effects were not seen following CNIH3 knockdown in males. Several studies have reported that female rats perform worse than males on spatial tasks such as the Morris water maze, which is thought to be due to decreased phosphorylation of s845 on GluA1 [50, 150]. Since CNIH3 increases the insertion of GluA1-containing AMPARs, these findings suggest that the functional role of CNIH3 in females may be crucial for short-term spatial memory, whereas it may not have a significant impact on this process in males.

### Transmembrane AMPAR regulatory proteins (TARPs)

TARPs are auxiliary proteins (γ − 1 to γ − 8) that modulate AMPAR trafficking, localization, and function. Stargazin, or TARP γ − 2, and TARP γ − 8 are two well-studied subtypes that regulate AMPAR surface expression through their interaction, via their PDZ domain, with proteins like PSD-95 (Fig. 1) [83, 151]. As previously mentioned, TARPs interact with all AMPAR subunits and regulate their functional properties, such as slowing deactivation and desensitization of GluA1-containing AMPARs which is critical for synaptic plasticity [59, 152–158]. In male mice, TARPγ − 8 also impaired contextual and cue-induced freezing during fear conditioning, but it is unknown whether it does the same in females, as it has not been explored [156]. Similar to the other proteins, drug exposure can alter the function of TARPs, thereby disrupting GluA1 trafficking and addiction-like behaviors. Cocaine sensitization-induced increases in GluA1-containing AMPARs are regulated by stargazin [159], and disrupting TARPγ − 8 causes attenuation of the acquisition of ethanol self-administration and cue-induced reinstatement, an effect that is not present during sucrose self-administration [160, 161]. These studies further highlight TARPs role in AMPAR trafficking and substance use disorder. However, only one of these studies included both sexes, but data from each sex were combined, which could potentially mask any potential sex differences [160]. Whether the role of TARPs is similar in both sexes remains to be determined.

## Conclusion

**Fig. 3:**
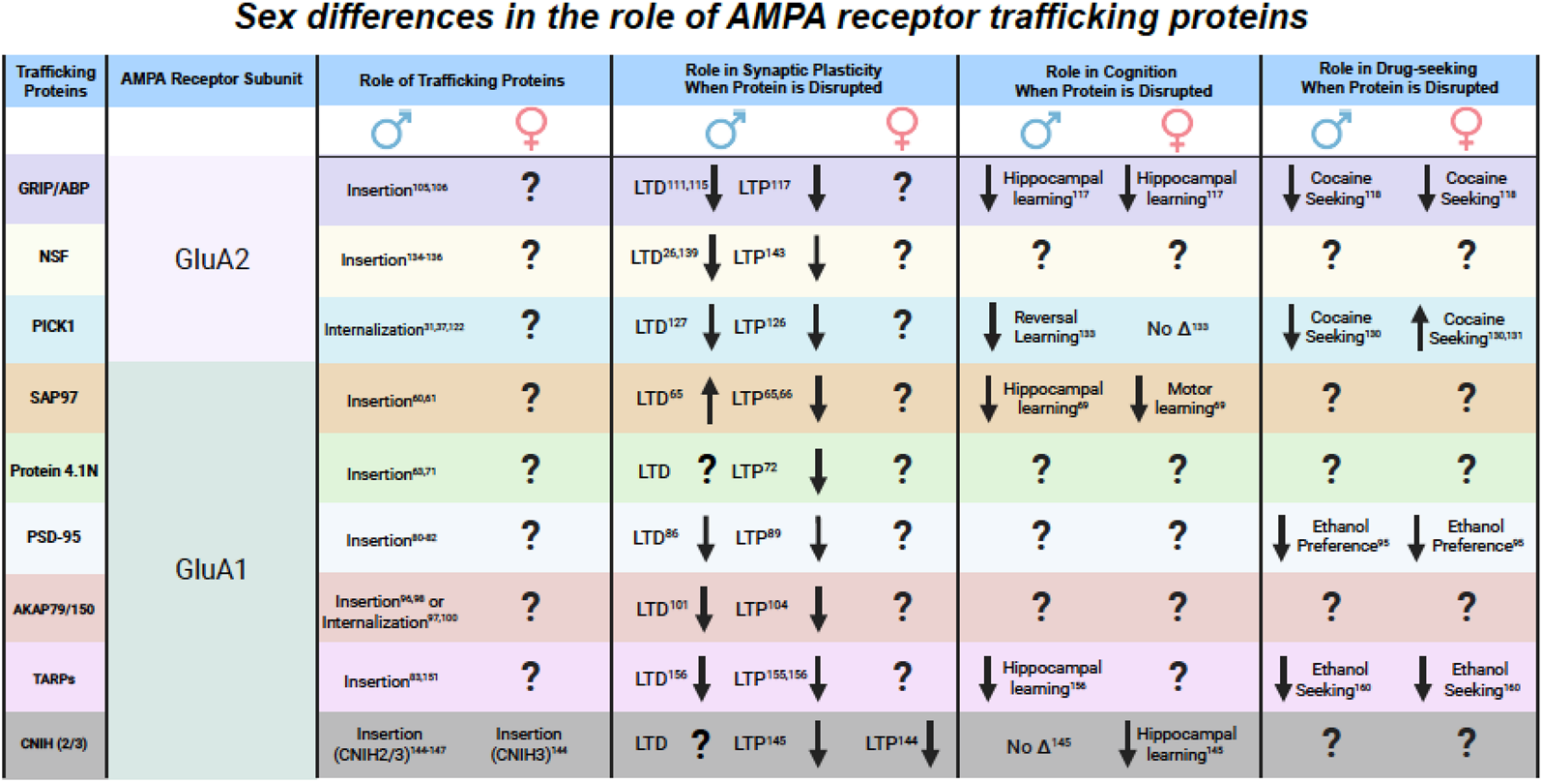
Summary table representing sex differences in the role of AMPAR trafficking proteins in trafficking, synaptic plasticity, cognition, and reward. Question marks indicate no research has been published on the topic. Overall, there is a need for more examination of the role of these trafficking proteins in females, as very little work has been done to determine if sex differences exist

Here, we showed evidence that, although heavily understudied, sex differences in the expression and function of trafficking proteins on glutamate receptor trafficking have been reported (Fig. 3). However, whether sex differences are present varies and depends on the specific trafficking protein, receptor, cell type, and brain region that is being investigated. In some cases, such as with GRIP and TARP, disrupting the protein causes identical behavioral effects in both sexes, regardless of the brain region in which they are disrupted, suggesting that their function in AMPAR trafficking might be similar across sexes. In contrast, most other proteins, such as PICK1, SAP97, and CNIH, exhibit sex differences across various behaviors. This suggests that the role of these proteins may differ between the sexes or different circuits or brain regions may mediate these behaviors in females compared to males. To date, there are no studies examining the effect of sex on the functional roles of NSF and AKAP79/150 in AMPAR trafficking, synaptic plasticity, and behavior.

Another layer to the story of sex differences in AMPAR trafficking proteins appears to be brain region specificity. Sex-specific cocaine seeking responses in the medial prefrontal cortex seem dependent on which trafficking protein is knocked down. When GRIP is knocked down, both sexes have attenuated cocaine seeking. In contrast, PICK1 KD produces a sex-specific response that appears to be mediated by ovarian hormones. In males, GRIP and PICK1 appear to have opposite functional roles that drive opposing behaviors during cue-induced reinstatement. This is not the case for females, and this suggests that PICK may play a different or additional functional roles in females. Within the prefrontal cortex, female rodents exhibit enhanced glutamatergic transmission and more GluA2-lacking AMPARs [162]. Therefore, the glutamate system in female mice may respond differently to manipulations of PICK1 expression. Additionally, PICK1 can bind many other proteins in the brain, and the binding affinities may be influenced by biological sex [125, 132, 163–165].

Much like the medial prefrontal cortex, sex-specific effects in the hippocampus on cognition are also dependent on which trafficking protein is knocked down. GRIP KD produces the same response in both sexes [117, 118]. However, GRIP’s role in synaptic plasticity in females remains unknown, and it is possible that these processes could differ. Similar to the sex-specific responses seen following prefrontal PICK1 KD [130], hippocampal PICK1 KD also produces sex-specific behavioral effects, suggesting that PICK1’s role in AMPAR trafficking in the hippocampus may also differ between males and females [133]. Knockdown of SAP97 also produces sex-specific responses, where males show reduced hippocampal learning, whereas females show deficits in motor learning [69, 70]. Here, the role of SAP97 in AMPAR trafficking may also be different across sexes. It is also entirely possible that AMPAR trafficking plays a sex-specific role in these behaviors. Studies examining the functional role of SAP97 in both sexes, using electrophysiological or molecular biological approaches, are essential to understanding its function. Both SAP97 and 4.1 N work alongside each other to facilitate GluA1 insertion, it is still unknown whether hippocampal 4.1 N also influences motor learning in females. Additional research on 4.1 N’s behavioral role is necessary to clarify the reported differences. Overall, current information is insufficient to fully understand these proteins’ activities, though some conclusions can be drawn. To better understand how AMPAR trafficking proteins differ between males and females, further behavioral and physiological studies are required.

While each of these proteins has specific roles, they operate together, sometimes redundantly, to maintain essential processes that are stable and adaptable to changing conditions. However, the way in which some of these proteins function between sexes differ across disease models. It is evident from these studies that considering sex as a biological factor is crucial when examining glutamate receptor trafficking and the associated proteins. This understanding could be key for gaining insights into learning processes and psychiatric diseases such as schizophrenia, AD, MDD, and SUDs, as these conditions affect males and females differently. However, little work has been done to investigate potential sex differences in trafficking protein expression and activity in either baseline or in a diseased state. The majority, if not all, of the preclinical research on the glutamate system has focused on males or conducted in cell cultures, therefore limiting its applicability to females. Due to the presence of similar trafficking proteins in both males and females, assumptions have been made that the trafficking mechanisms must be consistent across each sex. However, this assumption is flawed, as there are many sex differences within the glutamate system, including some of the trafficking proteins discussed in this review [41, 49, 50]. The current understanding of trafficking proteins in females is limited, and most research on the topic investigates behavioral phenotypes in disease models.

The impact of sex on AMPAR trafficking activity and synaptic activity remains largely unexplored. Future studies are needed to understand the exact mechanisms of these effects under basal conditions or in response to stimuli, as most research has been conducted in disease models. Before fully understanding the impact of these proteins in disease models, we must first characterize possible baseline physiological, molecular, genetic, and behavioral sex differences. This would help determine whether the observed sex differences in trafficking proteins are driven by gonadal hormones, by higher sex-dependent organizational effects, or by distinct sex-dependent functional roles of the protein. This would be informative for understanding how these proteins are affected in a diseased state or in response to stimuli. Likewise, these differences could enhance our understanding of why females are more or less susceptible to various psychiatric diseases or abusing certain substances. Therefore, we must continue investigating the role of sex in behavior and biological mechanisms, especially if we want to find novel avenues of research for developing therapeutics for diseased states.

## Data Availability

No datasets were generated or analysed during the current study.
